# Stop Saying That It Is Wrong! Psychophysiological, Cognitive, and Metacognitive Markers of Children’s Sensitivity to Punishment

**DOI:** 10.1371/journal.pone.0133683

**Published:** 2015-07-28

**Authors:** Maria Luz Gonzalez-Gadea, Anouk Scheres, Carlos Andres Tobon, Juliane Damm, Sandra Baez, David Huepe, Julian Marino, Sandra Marder, Facundo Manes, Sofia Abrevaya, Agustin Ibanez

**Affiliations:** 1 Institute of Cognitive Neurology (INECO), Buenos Aires, Argentina; 2 National Scientific and Technical Research Council (CONICET), Buenos Aires, Argentina; 3 UDP-INECO Foundation Core on Neuroscience (UIFCoN), Faculty of Psychology, Diego Portales University, Santiago, Chile; 4 Behavioural Science Institute, Developmental Psychology, Radboud University, Nijmegen, The Netherlands; 5 Grupo de Neurociencias de Antioquia, Facultad de Medicina, Universidad de Antioquia, Medellin, Colombia; 6 Grupo de Neuropsicología y Conducta, Facultad de Medicina, Universidad de Antioquia, Medellin, Colombia; 7 University of Leipzig, Leipzig, Germany; 8 Laboratory of Cognitive and Social Neurosciences, Universidad Diego Portales, Santiago, Chile; 9 Laboratorio de Neuroimágenes, Universidad Nacional de Córdoba, Cordoba, Argentina; 10 Comisión de Investigaciones Científicas de la Provincia de Buenos Aires y Facultad de Psicología (UNLP), La Plata, Argentina; 11 Centre of Excellence in Cognition and its Disorders, Australian Research Council (ACR), Randwick, New South Wales, Australia; 12 Universidad Autónoma del Caribe, Barranquilla, Colombia; University of Bologna, ITALY

## Abstract

Neurodevelopmental evidence suggests that children’s main decision-making strategy is to avoid options likely to induce punishment. However, the cognitive and affective factors contributing to children’s avoidance to high punishment frequency remain unknown. The present study explored psychophysiological, cognitive, and metacognitive processes associated with sensitivity to punishment frequency. We evaluated 54 participants (between 8 and 15 years old) with a modified Iowa Gambling Task for children (IGT-C) which included options with varying long-term profit and punishment frequencies. Skin conductance responses (SCRs) were recorded during this task. Additionally, we assessed IGT-C metacognitive knowledge, fluid intelligence, and executive functions. Participants exhibited behavioral avoidance and high anticipatory SCRs to options with high frequency of punishment. Moreover, age, IGT-C metacognitive knowledge, and inhibitory control were associated with individual differences in sensitivity to punishment frequency. Our results suggest that children’s preference for infrequently punished decisions is partially explained by psychophysiological signals as well as task complexity and development of cognitive control.

## Introduction

Ferb, an eleven-year-old boy, is completing an e-tutorial in preparation for a math exam. He is frustrated by the visual and auditory cues indicating errors in the tutorial. Also, he is eager to finish the exercises quickly and play his favorite videogame. However, Ferb knows that if he obtains a passing mark in the exam, he will have two months of vacations.

Decision-making requires making trade-offs such as suppressing the need for immediate reward and tolerating punishments or errors in the short term, in order to achieve long-term goals. Neurodevelopmental studies suggest that children’s main decision-making strategy is to avoid options with a high frequency of punishment [1–6]. However, the psychophysiological and cognitive processes contributing to sensitivity to punishment frequency remain unknown.

Decision-making has been evaluated with the Iowa Gambling Task (IGT) [7] and similar tests adapted for children [4,8]. In these tasks, participants have to win game money by selecting cards from four decks, which differ in the magnitude and frequency of gain and loss. There are two decks considered advantageous in the long run and also two disadvantageous decks. Both options also contained one deck with high punishment frequency and other with low probability of punishment. Most studies found that children do not make advantageous decisions in the IGT until late adolescence [8–12]. However, other reports have noted that children did not behave randomly and selected decks with infrequent punishment, despite the options’ long-term profit [2–4,6,13,14]. It is important to note that previous versions of the IGT feature important caveats for children assessment. This complex task involves several processing dimensions (amounts, frequencies, wins, losses). In addition, it demands mental calculations of costs and profits, which proved challenging for young children (e.g., operations with negative numbers).

Besides, implicit emotional processing during IGT performance has been extensively reported in adults through psychophysiological markers, such as skin conductance response (SCR) [15,16–18]. These studies have shown that participants exhibit SCR changes in response to the outcome of their choices (win versus loss). Remarkably, healthy adults show an anticipatory SCR before selecting a disadvantageous option. These signals have been interpreted as an index of emotional arousal and implicit processes underlying advantageous decision making. However, evidence of psychophysiological processes associated with IGT performance in children is scarce. Crone and van der Molen [2] reported that anticipatory SCR to disadvantageous options is absent in children (but see [19]) although present in adolescents (up to 16 years old) when choosing options with high punishment frequency. We suggest that the complexity of the IGT involving four options and different dimensions makes it difficult to disentangle the effects of long-term profit and punishment frequency on children’s SCR.

Several developmental studies have suggested a relation between IGT performance and cognitive abilities such as fluid intelligence (FI) and executive functions (EFs) [3,9]. For instance, cognitive control is one of the EFs which naturally correlates with children’s ability to make advantageous choices. However, some reports failed to find associations between these processes [10,11,20–22]. These inconsistencies could be explained by differential strategies during IGT performance [3,6,23]. Furthermore, to our knowledge, no study has assessed the involvement of FI and EFs in children’s sensitivity to punishment in the IGT.

Finally, participants’ task rules comprehension and option payoffs (explicit task-relevant knowledge) [24,25] as well as metacognitive knowledge [26,27] have been positively correlated with performance in adults. However, no previous study has explored whether children’s metacognitive knowledge is associated with performance on decision-making tasks.

### Aims and predictions

This study explored the role of psychophysiological responses, cognitive abilities (FI and EFs), and IGT metacognitive knowledge in children’s sensitivity to punishment. To this end, we designed a simplified IGT adapted for children, where the four original decks were presented in two task versions with different difficulty level. In both versions we tested the influences of punishment frequency during decision making. In the easy version, children selected between an advantageous deck (AD) with low punishment frequency (AD-L) and a disadvantageous deck (DD) with high punishment frequency (DD-H). We expected that participants would easily identify the AD in this version due to frequency bias. Children also performed a more difficult (hard) version in which the AD included high punishment frequency (AD-H) while the DD was associated with low punishment frequency (DD-L). We predicted that participants would present difficulties to discriminate between both AD and DD in this version.

We also assessed SCRs prior to card selection (anticipatory SCR) and after feedback. We predicted that both SCR measures of implicit learning and post-feedback processing would reflect children’s preference for infrequent punishment. In addition, we expected both behavioral and psychophysiological measures of decision making to be associated positively with age [2]. Last, we explored whether demographics (age and gender), cognitive abilities (FI and EFs), and IGT-C metacognitive knowledge were associated with individual’s differences in sensitivity to punishment.

## Material and Methods

### Participants

Fifty-nine participants, between eight and 14 years of age, were recruited from two private schools to participate in the study. Both schools were located in the same neighborhood of Buenos Aires City and featured students from middle to high socioeconomic status. As in other reports [28], some of them (7.75%) were excluded due to absent SCRs. The final sample included 54 (31 female) participants with a mean age of 11.13 (*SD* = 2.01). None of them reported a history of psychiatric or neurological disorders or were under psychopharmacological treatment. All participants provided a written informed assent, and a parent, next of kin, caretakers, or guardian gave written informed consent on behalf of the child enrolled in this study. These written informed consents follow the norms of the declaration of Helsinki. The study was approved by the Ethics Committee of the Institute of Cognitive Neurology.

### Instruments

#### IGT for children (IGT-C)

**Fig 1:**
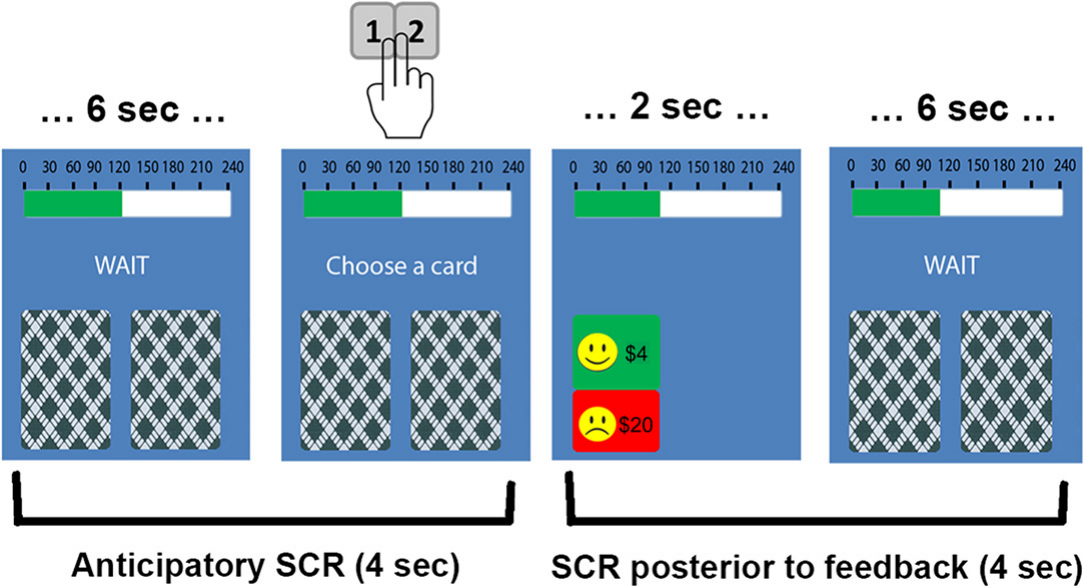
Trial sequence of the IGT-C. Each trial begins with a screen showing two decks and a “wait” message during 6 sec. Participants then select a deck by pressing 1 or 2 (second screen). Following response selection, an outcome screen shows the card selected (2 sec). After that, a new trial starts. The window of interest for SCR measures is shown below the screenshots. The example belongs to a card selected from de disadvantageous deck of the hard version.

We adapted the computerized four-deck IGT to design two versions suitable for children, with two decks each. Fig 1 shows an example of a trial sequence. Each trial began with the presentation of a stimulus for 6 seconds (sec), during which participants could ponder on their decision. A message then asked for a response. Participants took roughly between 0.5 and 2 sec to respond, without time pressure. After the response, the stimulus was replaced by a 2 sec outcome display. Thus, inter-trial intervals ranged from 8.5 to 10 sec.

Participants were instructed to select a card from either the left or the right deck by pressing 1 or 2 with the middle and index fingers of their dominant hand. Their goal was to maximize an initial capital ($120) represented by a money bar on the top of the stimulus display. Every time a card was selected, an outcome display revealed the back of the card depicting either a win or a loss. Winning feedback consisted of a green card with a happy face showing the amount earned. Loss feedback showed a card split down the middle: green with a happy face on the top and red with a sad face on the bottom (Fig 1). Immediately after feedback, the money bar was updated to reflect the balance or total amount won or lost in the trial. Thus, money bar represented the overall amount of winnings so far. Each version of the IGT-C included two decks differing in their long-term profit (AD and DD) and punishment frequency (high and low) (Table 1). Both versions contained an AD with small wins ($2) and a DD with high wins ($4). Every card from both decks includes a win, as a result the frequency of reward remains constant in the task (100%). However, the magnitude and frequency of punishment differed across decks and versions. In the easy version, the punishment frequency was low (20%) for the AD-L and high (50%) for the DD-H. In the hard version, punishment frequency between decks was inverted (AD-H and DD-L).

**Table 1:** Distribution of the reward and punishment across the decks and versions of the IGT-C.

		Reward		Punishment	
		Amount	Frequency	Amount	Frequency
		$	%	$	%
**Easy Version**	AD-L	2	100	6–4	20
	DD-H	4	100	8-10-12	50
**Hard Version**	AD-H	2	100	1-2-3	50
	DD-L	4	100	20–30	20

* AD-L: Advantageous deck with low punishment frequency

DD-H: Disadvantageous deck with high punishment frequency; AD-H: Advantageous deck with high punishment frequency; DD-L: Disadvantageous deck with low punishment frequency.

Participants were blind to both the distribution of reward and punishment between decks and the number of trials in each version (100). Every 20 trials, a black screen reading “break” indicated that participants could rest for a while, before resuming the task by pressing the spacebar. Participants were informed that they would receive chocolates after task completion, according to the accumulated money: one for less than $120, two for a profit between $120 and $180, and three for more than $180. All participants performed both versions which lasted approximately 15 minutes each. During a pilot study we observed similar performance when we counterbalanced the presentation order of the easy and hard versions (see S1 Text). However, children were less motivated and perceived poor performance when they played the hard version first. For this reason, in the current study we presented the easy version followed by the hard version. In both versions, we counterbalanced the left and right distribution of the AD and DD. Following classical IGT analysis [7], we measured the number of cards selected for each deck and we calculated a net score for each version by subtracting the number of cards from the AD minus the DD.

#### SCR recordings and processing

SCR were recorded during the IGT-C using a constant voltage (0.5 V) with Ag/Ag-Cl electrodes attached to the distal phalanx surfaces of the middle and index fingers of the non-dominant hand. The SCR was amplified through a BIOPAC system (MP100) and sampled on AcqKnowledge software at a rate of 200 Hz.

SCR data was analyzed using Matlab 7.1 and Ledalab toolbox (http://www.ledalab.de/). To decompose the raw skin conductance signal into phasic components, we used a discrete decomposition analysis [29]. The SCR area under the curve was calculated for two windows of interest: (1) anticipatory SCR for the 4 sec preceding response selection, and (2) post-feedback SCR for the 4 sec after feedback onset (Fig 1). SCRs were considered significant if higher than 0.01 μS [29].

#### IGT-C metacognitive knowledge

We developed a structured questionnaire to assess the participants’ metacognitive knowledge, including understanding of task variables and motivation during the task. First, we asked how enjoyable the task was on a 4-point scale (motivation). Second, we evaluated the participants’ abilities to calculate their net score per trial (the amount of win minus the amount of loss in a trial). We showed six examples of trials that implied a negative net score (four items), a positive payoff (one item) and a balance between win and loss (one item). Participants had to indicate which of three possible answers corresponded to the net score shown. Finally, we included a question to check understanding of the long-term profit of both decks and index knowledge of the task. The questionnaire was completed at the end of the IGT-C (see details in S2 Text).

#### Cognitive assessment

We evaluated the participants’ cognitive profile using measures of FI and EFs. A detailed description of tasks and measures is provided in S3 Text.

FI was evaluated with the Raven’s Progressive Matrices Test (RPMT) [30]. EFs were assessed through several instruments: (1) sub-tests from the Wechsler Intelligence Scale for Children, fourth edition (WISC IV) [31] (digit span, arithmetic, and letters and numbers), to assess working memory; (2) the child’s version of the Hayling test, to verbally assess response inhibition [32,33]; (3) the Trail Making Test, to assess attention (TMT-A) and set-shifting (TMT-B) [34]; and (4) the Battersea Multitask Paradigm (BMP), an ecological measure of EFs which taps several executive domains [35]. Participants had to complete three games (fruit sorting, caterpillar coloring, and counter sorting) within a lapse of three minutes while following four constrained rules (see S3 Text). Children were instructed to generate a plan before starting (planning abilities). BMP performance was evaluated considering number of tasks attempted (set-shifting), strategy performance (strategy formation), and rule-breaking behavior or number of errors (inhibitory control).

### Data analysis

A repeated measures analysis was used to assess performance and psychophysiological responses in the IGT-C according to: (1) long-term profit of the options (AD vs. DD) in each version, and (2) punishment frequency across versions (high vs. low). An ANOVA test was employed to compare the number of cards selected. Given that SCR measures do not satisfy the assumption of normality, a non-parametric Wilcoxon signed-rank test was used to perform SCR comparisons. In addition, we performed correlation analyses between age and both behavioral and psychophysiological measures. Pearson’s coefficient was used for parametric variables while Spearman’s ranks test was used for non-parametric measures. The significance of all correlations was corrected for multiple comparisons using the Sidak method (adjusted α level after correction of .01). To explore individual differences in sensitivity to punishment frequency, we split participants in groups *a posteriori* according to their IGT-C performance. Positive net score showed that most card selections were from the AD, while negative net score evidenced the preference for options from the DD. All participants obtained a positive net score in the easy version; while because of the frequency bias, negative and positive scores were observed in the hard version (see Fig 1B). Therefore, subjects who obtained a negative net score in the hard version were considered participants with high sensitivity to punishment frequency. On the contrary, participants who obtained positive net score on the hard version were categorized as subjects with low sensitivity to punishment frequency. Groups were compared along the variables of gender (chi square test) and age (student’s *t*-test). Given that sensitivity to punishment frequency is highly associated with age [1,3,6], we included this variable as a covariate in an ANCOVA analysis to compare the groups’ metacognitive and cognitive profiles. Both significant group differences before and after covariance are reported.

## Results

### Are children sensitive to punishment frequency?

**Fig 2:**
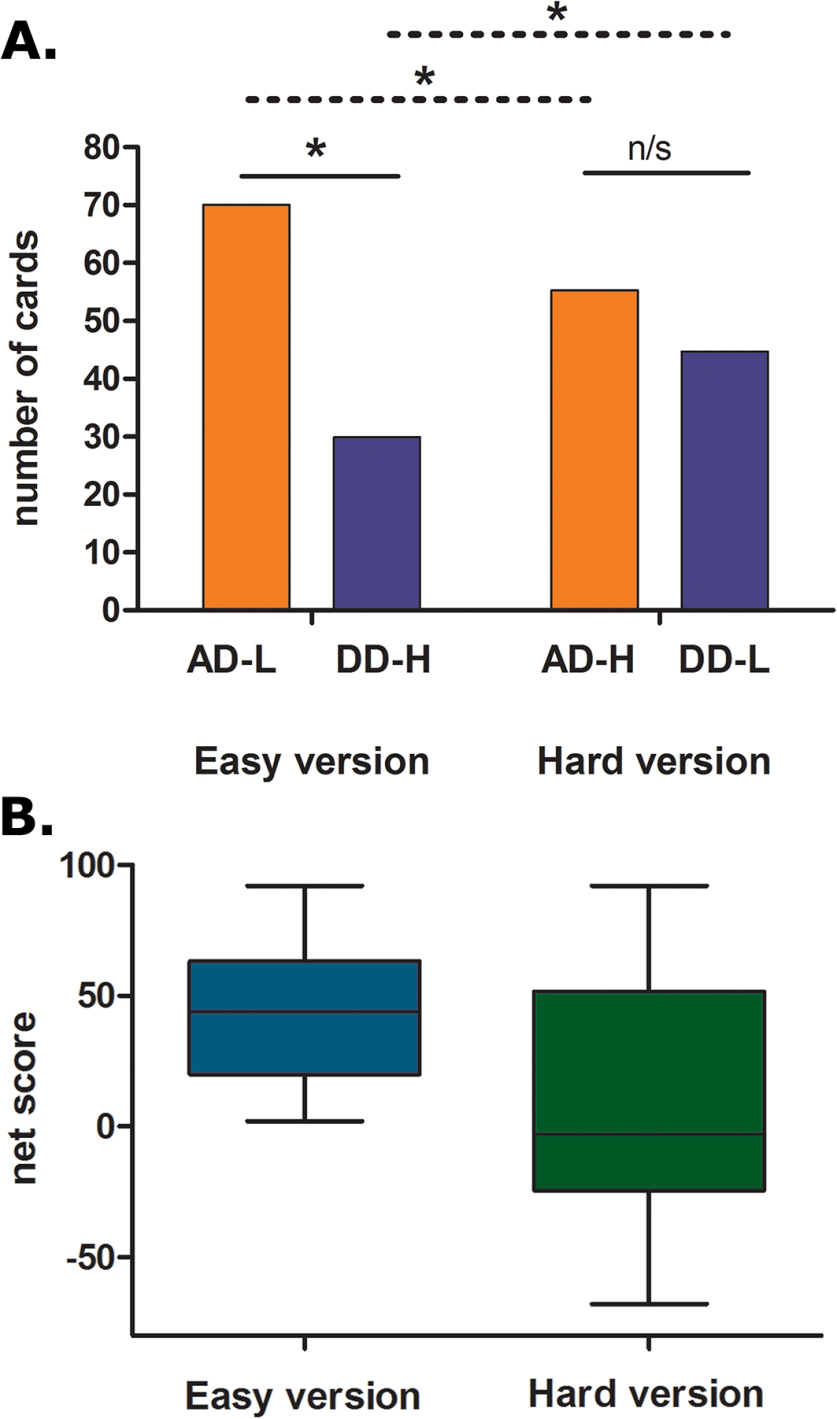
IGT-C number of cards selected per decks. **(A)** Mean number of cards selected from each deck of each version. (B) Box plots of net scores of each version.

We performed repeated measures analysis between the number of cards selected from the AD and DD. In the easy version, participants made significantly more choices from the AD-L than the DD-H (*F*_(1, 53)_ = 122.01, *p =* .001) (Fig 2). However, no differences between decks were observed in the hard version (*F*_(1, 53)_ = 2.87, *p* = .095). A comparison of decks with different punishment frequency showed that children significantly selected more cards from decks with low than high punishment frequency in both ADs (AD-L > AD-H; *F*_(1, 53)_ = 19.88, *p =* .001) and DDs (DD-L > DD-H; *F*_(1, 53)_ = 19.88, *p =* .001) (see S1 Table). In addition, we performed correlations between age and net scores. Age was significantly associated with performance in both version (easy version: *r* = 0.39, *p =* .010; hard version: *r* = 0.54, *p =* .000).

These results evidence that participants discriminated between ADs and DDs only when the AD was associated with infrequent punishment (easy version). However, they failed to do so when the AD had high punishment frequency (hard version). Furthermore, comparing decks according their punishment frequency, we observed that children avoided options with high probability of punishment (DD-H<DD-L and AD-H<AD-L). Finally, age was significantly associated with performance in all decks from both versions, suggesting that advantageous decision making improves with age.

### Does anticipatory SCR discriminate between options with different punishment frequency?

**Fig 3:**
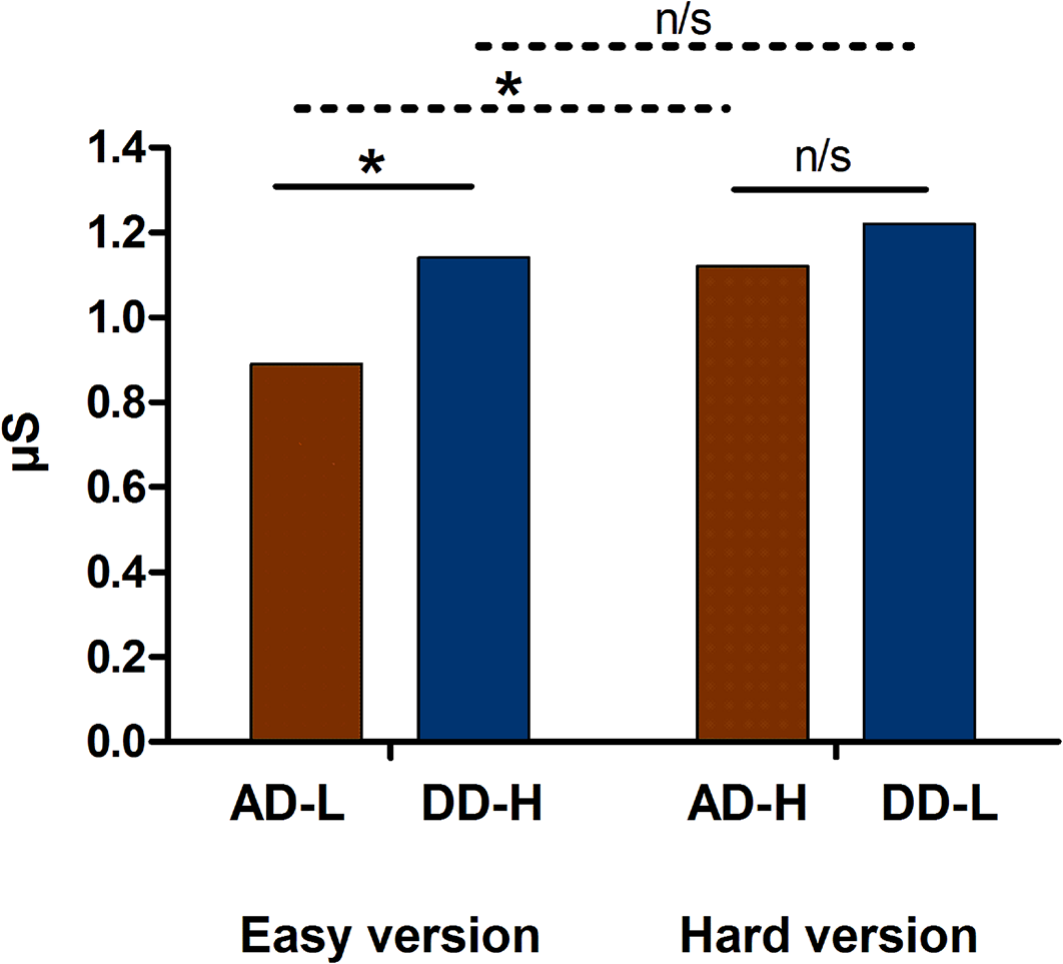
Anticipatory SCR. Mean of participants’ anticipatory SCRs (area under the curve) for each deck of the IGT-C, and comparisons between options.

Non-parametric pair-wise comparisons were used to assess anticipatory SCR between (i) ADs and DDs and (ii) decks with high and low punishment frequency (Fig 3). In the easy version, significantly higher anticipatory SCRs were observed in DD-H compared to AD-L (*z* = 1.46, *p* = .049). Instead, the hard version yielded no significant differences between decks (*z* = 0.23, *p* = .814). In addition, significant differences were observed between decks with high and low punishment frequency. Participants showed higher anticipatory SCR in AD-H than in AD-L (*z* = 2.91, *p* = .003). However, no significant differences were observed between DD-H and DD-L (*z* = 0.02, *p* = .978) (see details in S1 Table).

Furthermore, we used Spearman’s test to assess the relationship between age and anticipatory SCR measures. No significant associations were found between these variables (see S2 Table).

Consistent with behavioral responses, these results show that anticipatory SCR discriminated between AD and DD only when the AD was associated with infrequent punishment (easy version). In addition, anticipatory SCR differentiated between options with high and low punishment frequency for ADs but not for DDs. None of these SCR indexes were associated with age.

### Does SCR after feedback discriminate between options with different punishment frequency?

**Fig 4:**
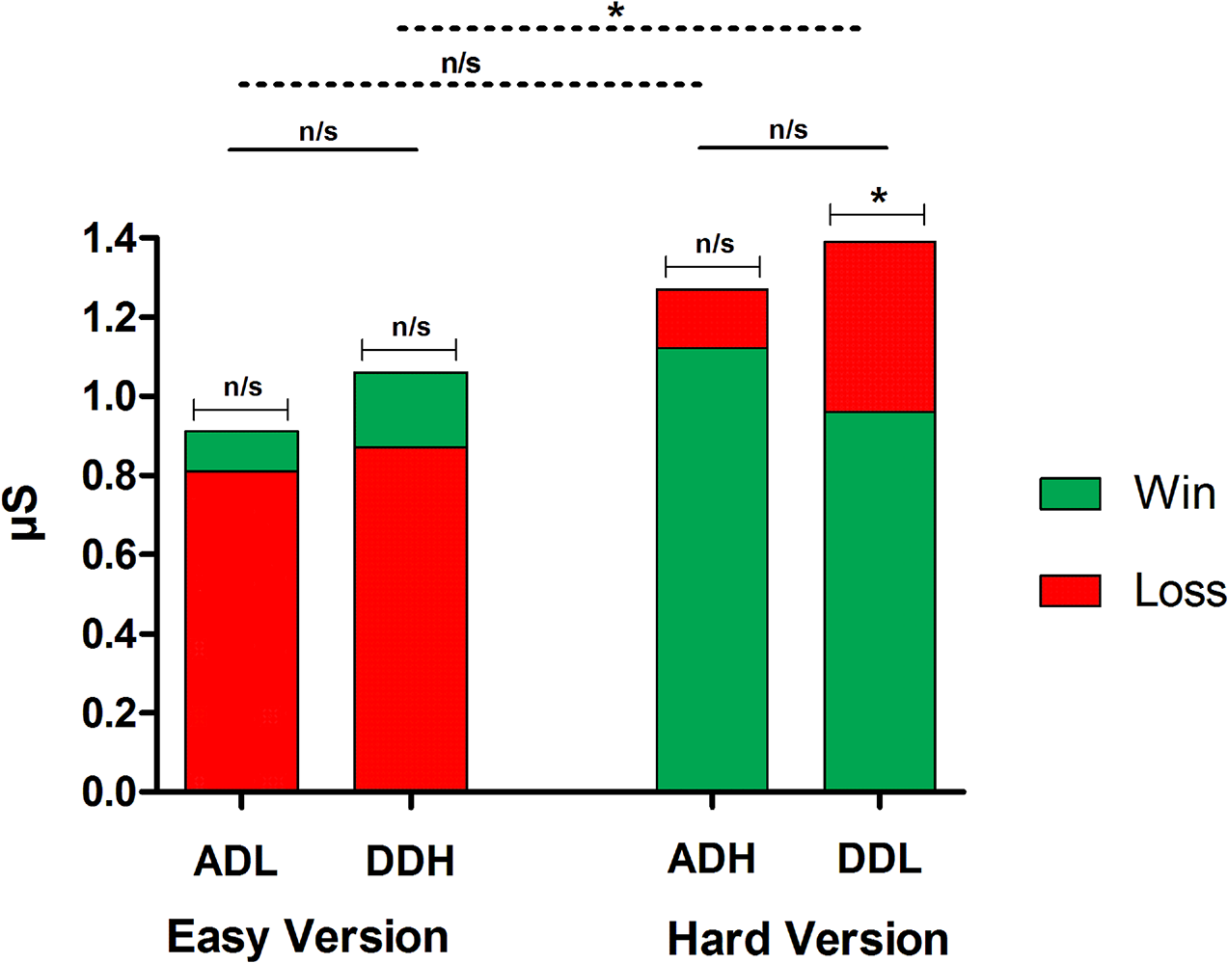
SCR after feedback. Mean of participants’ SCRs after feedback (area under the curve) for each deck of the IGT-C, and comparisons between options. Bars of win and loss feedback are superimposed in each deck.

Fig 4 shows SCR after feedback. First, comparisons between SCR after win and SCR after loss were performed for each deck. In the easy version, no significant differences were observed in either deck (AD-L: *z* = 0.96, p = .332; DD-H: *z* = 1.21, *p* = .223). In the hard version, although no significant differences were found in the AD-H (*z* = 0.30, *p* = .761), SCR after loss was significantly higher than SCR after win in the DD-L (*z* = 3.43, *p* = .000).

For comparisons between decks, we calculated the difference between SCR after loss and SCR after win for each deck as a composite measure for SCR after feedback (see details in S1 Table). The comparison of decks with different long-term profit (AD versus DD) revealed no significant differences in either the easy (*z* = -.13, *p* = .896) or the hard (*z* = -.85, *p* = .393) version. As regarding decks with different punishment frequency, no significant differences were found between AD-L and AD-H (*z* = -1.85, *p* = .063). However, SCR after feedback was significantly higher for DD-L (hard version) than DD-H (easy version) (*z* = -2.31, *p* = .021).

Lastly, we performed correlations between age and measures of SCR after feedback. No significant associations were found between these variables (see S2 Table).

In sum, results show that post-feedback SCR was not modulated by punishment frequency. However, SCR modulations for win/loss were observed in the DD-L. Similarly, SCR after feedback was significantly higher for DD-L than for DD-H. Remarkably, the former deck contained the highest magnitude of losses in the task. Thus, SCR after feedback seems sensitive to unexpected high loss rather than punishment frequency. Finally, none of these psychophysiological signals was related to age, suggesting that SCR after feedback was similar across the sample.

### Cognitive and metacognitive processes associated with individual differences in sensitivity to punishment frequency.

On the basis of performance in the hard version, participants were categorized as either more or less sensitive to punishment frequency (see criteria group formation in Data analysis). Table 2 shows group comparisons along the variables of gender and age (demographics), IGT-C metacognitive knowledge, and cognitive abilities.

**Table 2:** Means, SDs and group comparisons between participants with high and low sensitivity to punishment frequency.

		High sensitivity to Punishment Frequency (N = 28)	Low sensitivity to Punishment Frequency (N = 26)	Group differences *	Ancova (age)*
**Demographics**	** **	** **	** **	** **	** **
	Gender (male:female)	9:19	14:12	.143	n.a
	Age	10.21 (1.75)	12.12 (1.84)	.000	n.a
**Cognitive Assesment**	** **	** **	** **	** **	** **
	RPMT	34.78 (7.41)	37.88 (5.98)	.098	.870
	Digit span	14.39 (2.02)	15.85 (3.94)	.091	.439
	Letters and numbers	15.11 (4.57)	17.50 (3.82)	.043	.580
	Aritmethic	21.93 (4.66)	24.92 (3.83)	.009	.453
	Hayling test	5.00 (4.17)	4.19 (4.67)	.505	.844
	TMT-A	25.78 (8.38)	23.32 (8.94)	.305	.918
	TMT-B	75.96 (33.58)	64.46 (29.33)	.187	.408
	BMP: Planning	6.29 (2.88)	6.15 (2.74)	.864	.647
	BMP: Task attemped	2.54 (0.74)	2.72 (0.46)	.290	.979
	BMP: Strategic performance	7.36 (2.51)	9.42 (2.73)	.006	.175
	BMP: Rule breaking	1.77 (1.68)	0.89 (1.23)	.032	.003
**Metacognitive knowledge of the IGT-C**	** **	** **	** **	** **	** **
	Motivation	3.54 (0.51)	3.38 (0.64)	.338	.753
	Calculation	3.96 (2.05)	5.85 (0.54)	.000	.002
	Knowledge	1.17 (0.9)	1.77 (0.65)	.009	.031

* For group comparisons a one-way Anova test was used except for gender (chi-square test). The Ancova test was used to compare cognitive and metacognitive measures, using age as a covariate.

RPMT: Raven’s Progressive Matrices Test; TMT: Trail Making Test; BMP: Battersea Multitask Paradigm.

#### Demographics

No significant group differences were observed in gender (*x* ^2^ = 2.14, *p* = .143). However, significant differences were found in age (*F*_(1,52)_ = 15.14, *p* = .000). Participants with high sensitivity to punishment frequency were younger than individuals with low sensitivity to punishment.

#### IGT-C metacognitive knowledge

We compared group differences in motivation, calculation, and knowledge of the IGT-C. Relative to children with low sensitivity to punishment frequency, participants with high sensitivity to punishment presented significantly lower scores in calculation (*F*_(1, 52)_ = 20.62, *p* = .000) and task knowledge (*F*_(1, 52)_ = 7.47, *p* = .009). These significant differences remained after adjusting by age as a covariate (see Table 2). No significant differences between groups were observed in motivation.

#### Cognitive assessment

Children with high sensitivity to punishment obtained less scores in two working memory subtest (Arithmetics: *F*_(1, 52)_ = 7.34, *p* = .009 and Letters and numbers: *F*_(1, 52)_ = 4.32, *p* = .043). However, these significant differences disappeared after adjusting by age (Arithmetics: *F*_(1, 51)_ = 0.57, *p* = .453; Letters and numbers: *F*_(1, 51)_ = 0.31, *p* = .580). No significant group differences were observed in FI, verbal inhibition, set-shifting, and most of the measures from the BMP (see Table 2). However, significant differences were found in the BMP’s rule breaking score (inhibitory control): children with high sensitivity to punishment made more errors (*F*_(1, 52)_ = 4.83, *p* = .032). These differences remained significant after adjusting by age as a covariate.

Overall, these results show that age, IGT-C metacognitive knowledge, working memory, and inhibitory control were associated with high sensitivity to punishment frequency. However, after adjusting by age, only IGT-C metacognitive knowledge and inhibitory control were the measures that differentiated children with high and low sensitivity to punishment.

## Discussion

In this study we explored the influence of psychophysiological, metacognitive, and cognitive variables in children’s sensitivity to punishment frequency during decision-making tasks. Our results showed increased anticipatory psychophysiological responses to most of the options involving high frequency of losses. In addition, age, IGT-C metacognitive knowledge, and inhibitory control were associated with individual differences in sensitivity to punishment frequency.

By using a modified IGT suitable for children, this study demonstrated that participants between 8 to 14 years-old develop anticipatory psychophysiological signals that accompany their preference for advantageous options with infrequent punishment. Although similar psychophysiological patterns was previously reported only in adolescents up to 16 [2], our study extended this finding to younger children. Also, this is the first developmental study demonstrating that metacognitive knowledge and inhibitory control also play a role on participants’ sensitivity to punishment frequency. Taken together, our data indicate that children’s preference for infrequent punishment is partially explained by psychophysiological signals as well as task complexity and cognitive control.

### Sensitivity to punishment frequency: Behavioral correlates

Developmental studies suggest that children are not able to consider the long-term consequences of their decisions until late adolescence [1–4,8–12]. Similarly, our results showed that performance in the IGT-C was associated with age. However, children have been shown to prefer options with infrequent punishment [1,3–6]. In our task, participants selected advantageously only when the AD featured infrequent punishment (easy version), but they failed to do so when the AD was associated with high punishment frequency (hard version). These results suggest that the ability to taking into consideration the choice’s long-term benefit decreased when it was associated with a high punishment frequency. Our findings are in line with previous reports [1,3–5,14,36] and confirm that children have a bias towards infrequent punishment.

Note, that in the hard version DD-L was not preferred over AD-H. Thus, it is likely that children do not always prefer options with infrequent punishment. Instead, this frequency effect seems to bias children’s choices and induce a shift between advantageous and disadvantageous choices. We suggest that this profile could be an inability to take into account the future consequences, but it could also be an unwillingness to experience negative emotions in the short term. In other words, children want to avoid the immediate negative feeling associated with frequent punishment.

### Sensitivity to punishment frequency: Psychophysiological correlates

Consistent with behavioral responses, anticipatory SCR was modulated by high and low punishment frequency. Only in the easy version, participants showed increased anticipatory SCR to the DD. In addition, higher SCR was observed in ADs depicting high punishment frequency rather than low frequency of losses. A previous report [2], has suggested that children perform like patients with VMPFC lesions because they show no psychophysiological responses prior to disadvantageous decisions. Conversely, our results show that children do exhibit anticipatory SCR as a correlate of behavioral performance–i.e., avoiding options with high punishment frequency. These findings are in line with theories that interpret anticipatory SCRs as covert emotional signals influencing decision-making [7,37]. We suggest that psychophysiological signals may be used as covert input to avoid high punishment frequency, which may explain children’s behavioral preferences.

However, within DDs, anticipatory SCR was not significantly modulated as a function of punishment frequency. Note that the DD with low punishment frequency (DD-L) represents the option with the highest loss magnitude in the task (Table 1). We propose that the high unexpected losses in this option may generate elevated anticipatory SCR, which attenuates the differences between both DDs.

Similarly, the highest post-feedback SCR was observed in the DD-L. Thus, this option produced the only significant modulation between win and loss. Thus, contrary to our expectations, SCR after feedback was modulated by the unexpected and high loss magnitude rather than punishment frequency.

Traditionally, high SCR after loss has been associated with a monitoring system indicating that performance should be adjusted on subsequent trials [38]. However, our participants persisted on selecting from the DD-L even after high SCR to losses. Similarly, Crone and van der Molen [2] found that increased SCR after loss in DDs was not different between participants with good and poor performance. These results suggest that SCR responses after high negative feedback could be associated with a general system that responds to aversive situations. Alternatively, these results could be framed within the Yerkes-Dodson law [39], which suggests that reinforcement signals that are too arousing will slow down subsequent performance rather than increase task focus.

In sum, our results show that high anticipatory SCR was partially used as an implicit signal accompanying the avoidance of options with high punishment frequency. Such a psychophysiological response may explain the children’s preference for infrequent punishment. Conversely, increased SCR after feedback was modulated by the unexpected high magnitude of losses. Thus, it may explain the persistence on selection from the disadvantageous option with low punishment frequency.

Lastly, contrary to our predictions, none of these psychophysiological measures was related to age. This finding suggests that both anticipatory SCRs modulation in response to punishment frequency and SCR after feedback in response to unexpected punishment magnitude is not directly associated with developmental changes, at least between 8 to 14 years-old.

### Cognitive and metacognitive processes associated with individual differences in sensitivity to punishment frequency

We explored whether demographic, IGT-C metacognitive knowledge, and cognitive variables would differentiate between children with high and low sensitivity to loss frequency. We found that participants with higher sensitivity to punishment frequency were younger, exhibited poorer metacognitive knowledge of the task, and had lower inhibitory control. We suggest that preference for infrequent punishment is associated with age, the complexity of the IGT, and with children’s ability to suppress prepotent responses.

First, as previously reported [1,3,4,14], we found that age was associated with reduced sensitivity to loss frequency. Second, this is the first developmental study to evaluate the influences of metacognitive knowledge on IGT performance. Although the groups did not differ in task motivation, participants with high sensitivity to punishment frequency reported poorer task-relevant knowledge and reduced calculation abilities. Previous reports have suggested that explicit knowledge about payoff structure during and after the IGT is an important predictor of adult performance [24,25,40]. Similarly, developmental studies [14,41] demonstrated that children learn to prefer advantageous options in the IGT when information about wins, losses, and probabilities are presented before the task starts. In the present study, we found that children with high sensitivity to punishment frequency exhibited less explicit knowledge about the decks’ long-term profit, as assessed by a post-task questionnaire. Thus, misunderstanding of the options’ future consequences may promote preference for infrequent punishment.

In addition, we found that children with high sensitivity to punishment frequency showed less ability to calculate the net score per trial in the IGT-C. This result may suggest that children’s high frequency bias could also be associated with reduced task understanding. Note that the task includes calculations with negative numbers–e.g., Fig 1 shows a trial with a win of $4 and a loss of $20, implying a total loss of $16. Given that negative numbers are usually introduced into the mathematics curriculum between fourth and sixth grade [42,43], it is not surprising that young children failed to perform such calculations. We also observed significant group differences in arithmetic and letters and numbers subtests of working memory which are highly correlated to math abilities [31]. However, these group differences disappeared after adjusting by age. Previous studies found no association between IGT performance and standardized measures of arithmetic skills [41]. These findings suggest that standardized arithmetic tasks may not be sensitive enough to assess children’s abilities to calculate the ongoing operations during the IGT.

In sum, we demonstrated that sensitivity to punishment frequency is influenced by the complexity of the IGT, which requires understanding and mental manipulation (calculations) of several task dimensions (gains, losses, and probabilities).

Finally, our results showed that children with low and high punishment sensitivity profiles did not differ across FI and most of the EFs measures. Similarly, previous reports [10,11,20,21,44–46], found no association between IGT performance and EF measures. However, children with high sensitivity to punishment frequency did make more errors in the BMP. Rule-breaking in multitasking settings has been attributed to poor inhibitory control in adults [47] and children [35]. In addition, low inhibitory control has been linked to high sensitivity to reward and punishment across development [48,49]. Since we compared groups with different tolerance to punishment frequency, it was not unexpected that inhibitory control became the EF which differentiated both groups. However, we did not find significant group differences in the other inhibition measure (the Hayling test), which resembles findings in other developmental studies [11,44]. The BMP is a complex, ecologically valid task that requires the inhibition of prepotent response in a real-life environment [35,50,51]. The Hayling test also demands response inhibition [52], but it could be solved with more basic rules (e.g., naming objects within the participant’s visual field). Hence, tolerance to high punishment frequency may be associated with cognitive control as assessed with more ecological executive tasks (such as the BMP).

### Limitations and future directions

First, we observed high variability in psychophysiological responses among participants. Futures studies should explore whether our results are replicated in larger samples. Likewise, the influence of metacognitive and cognitive variables on children’s decision-making should be investigated with more robust methods–such as structural equation modeling, which also requires an extended number of participants.

In addition, while our assessment of IGT-C metacognitive knowledge was based on a self-report questionnaire, children exhibit a dissociation between knowing and doing [53]. In other words, children sometimes fail to report knowledge (e.g., ability to identify the AD) that is present in their behavior (e.g., preferring the AD). Besides, our assessment of task knowledge does not reveal whether children identified long-term benefits or were focused on the amounts of win, loss, or punishment frequency. Future studies would benefit from using more objective measures, such as post-decision wagering [54], to assess the participants’ task knowledge. Similarly, the assessment of metacognition should be improved in future studies through an examination of decision confidence and knowledge previous to feedback presentation.

Lastly, we considered children which obtained positive performance on both easy and hard versions as participants with low sensitivity to punishment frequency. Those participants could also have employed other strategies (i.e., focusing on the amount of loss or the expected value of the outcomes). Futures studies should consider experimental paradigms designed to disentangle these strategies.

### Conclusions

In this study we developed a simplified IGT to explore the cognitive and psychophysiological processes associated with children’s sensitivity to punishment frequency. We suggest that this design helped to resolve some inconsistencies in findings reported previously. We found that high anticipatory SCR accompanies avoidance of high punishment frequency in most of the participants’ choices. We suggest that these implicit signals may bias children’s decision-making. In addition, we found that poor task metacognitive knowledge and low inhibitory control were associated with sensitivity to punishment frequency. This indicates that task complexity and cognitive control development may explain the observed preference for infrequent punishment.

Our findings have implications for both neurodevelopmental assessment and educational practice. First, developmental studies should control for task complexity and the children’s scholastic learning–e.g., the ability to solve arithmetic calculations. Otherwise, the children’s performance may be misinterpreted in the light of adult tasks and models [55]. Finally, sensitivity to punishment frequency may also have implications for educational practices involving continuous feedback to students. While feedback is crucial to improve and accelerate learning [56], we suggest that the conditions in which feedback is given may affect the students’ subsequent performance. In particular, feedback approaches highlighting the frequency of errors (as opposed to eventual achievements) may generate aversion to the tasks in question and promote disadvantageous decision-making.

## Supporting Information

S1 Dataset Data repository. (XLS)

S1 Fig Pilot study IGT-C. Mean number of cards selected from each deck and net score for the easy and hard version of the task.
(TIF)

S1 Table Mean and SDs of SCR measures for eack deck of the IGT-C. (DOCX)

S2 Table Correlations of IGT-C measures with age. (DOCX)

S1 Text Pilot study (DOCX)

S2 Text IGT-C questionnaire. (DOCX)

S3 Text Cognitive assessment. (DOCX)
