# Supplementary figures and images for: Stop Saying That It Is Wrong! Psychophysiological, Cognitive, and Metacognitive Markers of Children’s Sensitivity to Punishment
Source: PLoS One. 2015 Jul 28;10(7):e0133683. doi: 10.1371/journal.pone.0133683 (PMC4517808; doi:10.1371/journal.pone.0133683)

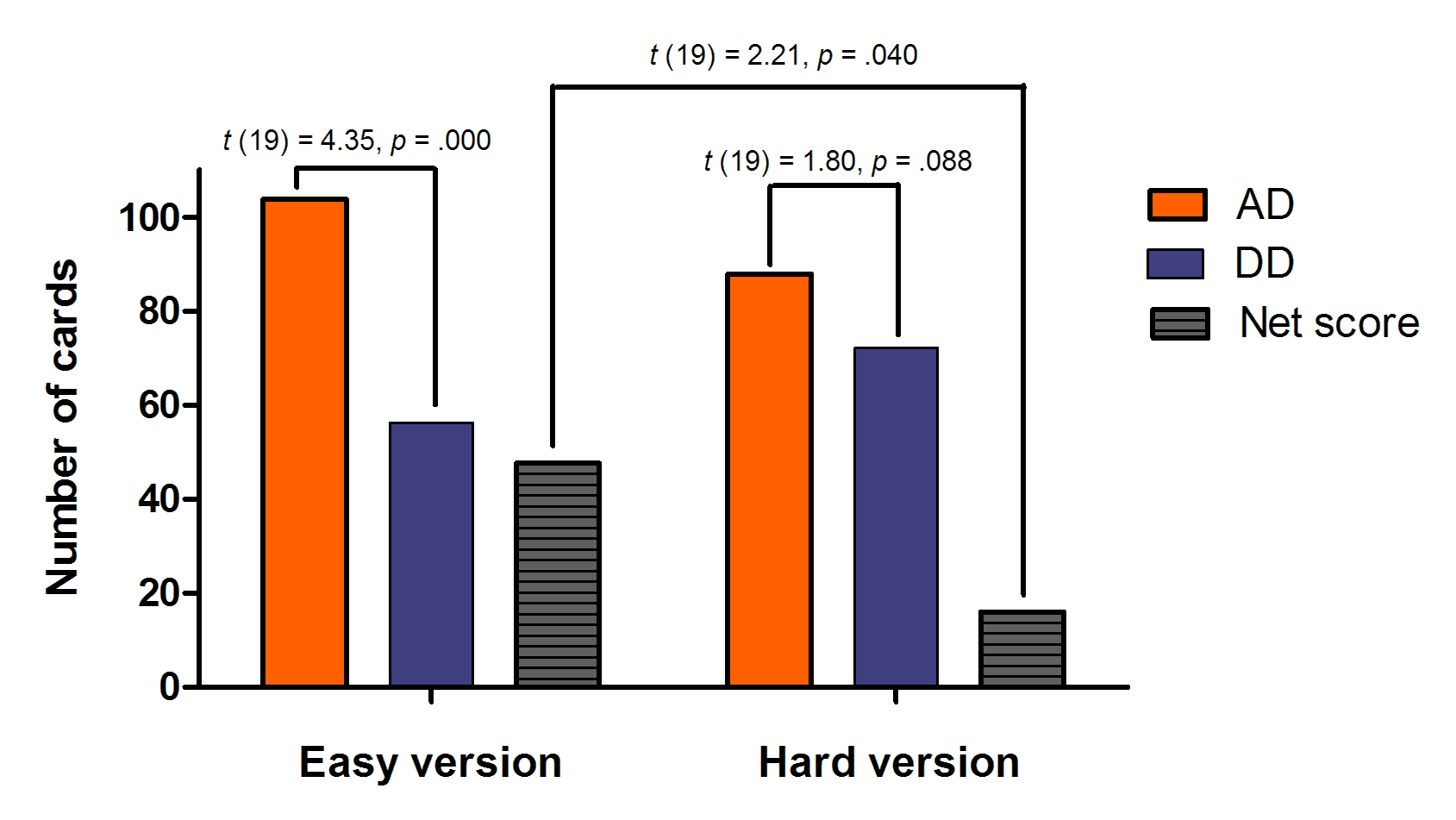

Supplement: S1 Fig — Mean number of cards selected from each deck and net score for the easy and hard version of the task. (TIF) [file pone.0133683.s002.tif]
