# Supplementary material for: Stop Saying That It Is Wrong! Psychophysiological, Cognitive, and Metacognitive Markers of Children’s Sensitivity to Punishment
Source: PLoS One. 2015 Jul 28;10(7):e0133683. doi: 10.1371/journal.pone.0133683 (PMC4517808; doi:10.1371/journal.pone.0133683)
Supplement: S1 Table — (DOCX) [file pone.0133683.s003.docx]

**S1 Table**

S2 Table: Mean and SDs of SCR measures for each deck of the IGT-C

|  | | Anticipatory SCR | SCR after win | SCR after loss | SCR after feedback * |
| --- | --- | --- | --- | --- | --- |
| Easy version | AD-L | 0.89 (0.46) | 0.91( 0.53) | 0.81 (0.52) | -0.10 (0.49) |
|  | DD-H | 1.14 (0.72) | 1.06 (0.69) | 0.87 (0.59) | -0.19 (0.71) |
| Hard version | AD-H | 1.12 (0.76) | 1.12 (0.70) | 1.27 (0.94) | 0.15 (0.87) |
|  | DD-L | 1.22 (0.98) | 0.96 (0.67) | 1.39 (1.07) | 0.33 (0.96) |

* Composite score: SCR after loss minus SCR after win. AD-L: Advantageous deck with low punishment frequency; DD-H: Disadvantageous deck with high punishment frequency; AD-H: Advantageous deck with high punishment frequency; DD-L: Disadvantageous deck with low punishment frequency.
