# Supplementary material for: Stop Saying That It Is Wrong! Psychophysiological, Cognitive, and Metacognitive Markers of Children’s Sensitivity to Punishment
Source: PLoS One. 2015 Jul 28;10(7):e0133683. doi: 10.1371/journal.pone.0133683 (PMC4517808; doi:10.1371/journal.pone.0133683)
Supplement: S2 Table — (DOCX) [file pone.0133683.s004.docx]

**S2 Table**

S3 Table: Correlations of IGT-C measures with age

|  | | Cards selected ^a^ | Anticipatory SCR ^b^ | SCR after feedback ^b^ |
| --- | --- | --- | --- | --- |
| Easy version | AD-L | .35* | .09 | -.11 |
|  | DD-H | -.35* | .05 | -.05 |
| Hard version | AD-H | .58* | .18 | .09 |
|  | DD-L | -.58* | .03 | -.01 |

a: Pearson's coeficient; b: Spearman's coeficient; * p > .01. AD-L: Advantageous deck with low punishment frequency; DD-H: Disadvantageous deck with high punishment frequency; AD-H: Advantageous deck with high punishment frequency; DD-L: Disadvantageous deck with low punishment frequency.
