# Supplementary material for: Stop Saying That It Is Wrong! Psychophysiological, Cognitive, and Metacognitive Markers of Children’s Sensitivity to Punishment
Source: PLoS One. 2015 Jul 28;10(7):e0133683. doi: 10.1371/journal.pone.0133683 (PMC4517808; doi:10.1371/journal.pone.0133683)
Supplement: S1 Text — (DOCX) [file pone.0133683.s005.docx]

**S1. Pilot study**

A pilot study was performed in order to determined whether version manipulation, number of trials and duration of the IGT-C were adequate. We assessed 20 children (13 males, 7 females) between 8 to 15 years of age (mean= 11.30 , SD= 2.22). Participants were randomly assigned to perform the easy version followed by the hard version (condition A) or the hard version followed by the easy version (condition B). No differences in age or gender were observed between these two groups (see Table below). An ad-hoc questionnaire (see section 1.1) and an interview were also used to evaluate children’s understanding of the task.

Table 1 shows the comparisons between children assigned to condition A versus those assigned to condition B. No significant differences between these groups were observed in the net score or in deck choices in the easy version. Only a significant tendency was observed in the net score of the hard version, where children who performed condition B exhibited poor performance than children who performed condition A. No significant differences were observed in deck choices in the hard version. We observed significant differences between both conditions in the questionnaire. Children who performed condition B reported significantly less motivation to play the game and perceived poor performance in the hard version than children who were assigned to condition A. No differences between conditions were observed in the perceived performance of the easy version.

Table 1. Mean, SD and group comparisons between participants who performed condition A and B

|  | | Condition A (N=10) | Condition B (N=10) | Group differences * | |
| --- | --- | --- | --- | --- | --- |
|  |  |  |  | t | P |
| Demographics | Age | 11.40 (2.17) | 11.10 (2.55 | .28 | .781 |
|  | Gender (males:females) | 7:03 | 6:04 | .64* | .500 |
| Easy version | AD | 95.00 (12.65) | 112.60 (30.50) | -1.68 | .109 |
|  | DD | 65.00 (12.65) | 47.40 (30.50) | 1.68 | .109 |
|  | Net score | 30.00 (25.33) | 65.20 (61.00) | -1.68 | .109 |
| Hard version | AD | 95.00 (18.87) | 80.80 (18.58) | 1.69 | .107 |
|  | DD | 65.00 (18.87) | 79.20 (18.58) | -1.69 | .107 |
|  | Net score | 32.50 (30.19) | -0.40 (33.90) | 2.05 | .055 |
| IGT-C questionnaire | Motivation | 3.56 (.527) | 3.00 (.535) | 2.15 | .048 |
|  | Perceived performance in easy version | 2.89 (.601) | 2.00 (.866) | .50 | .622 |
|  | Perceived performance in hard version | 2.33 (.866) | 2.23 (.835) | 2.53 | .022 |

* Chi-squuare test. Condition A: children performed the easy version followed by the hard version. Condition B: children performed the hard version followed by the easy version. AD: advantageous deck. DD: disadvantageous deck

These preliminary results show that the version’s order of presentation do not affect children’s performance in the easy version. However, the presentation of the hard version first (condition B) may negatively affect children’s performance in this version, as well as their motivation and perceived performance.

In all participants, we also contrasted the IGT-C performance in both easy and hard versions. S1 Fig. 1 shows that children obtained significant lower net scores in the hard compared to the easy version. Similarly, children discriminated between the AD and the DD in the easy version, while the difference between both options was abolished in the hard version, with no significant differences.

These preliminary results show that the punishment frequency might bias children's choices. Participants had a strong preference for AD compared to DD when it was associated with low punishment frequency (easy version), while this preference decreased when AD included high punishment frequency (hard version).

**Fig. 1: Pilot study IGT-C.** Mean number of cards selected from each deck and net score for the easy and hard version of the task.

**S1. 1. Pilot questionnaire**

1) This game was:

- Very exciting
- A little bit exciting
- Boring
- Very boring

2) How do you think you played in the first task? (easy task)

- Very well
- Well
- Bad
- Very bad

3) How do you think you played in the second task? (hard task)

- Very well
- Well
- Bad
- Very bad

**Scoring**

1) 4= Very exciting; 3= a little bit exciting; 2= boring; 1= very boring.

2 and 3) 4= Very well; 3= well; 2= bad; 1= very bad
