# Supplementary material for: Stop Saying That It Is Wrong! Psychophysiological, Cognitive, and Metacognitive Markers of Children’s Sensitivity to Punishment
Source: PLoS One. 2015 Jul 28;10(7):e0133683. doi: 10.1371/journal.pone.0133683 (PMC4517808; doi:10.1371/journal.pone.0133683)
Supplement: S2 Text — (DOCX) [file pone.0133683.s006.docx]

**S2. IGT-C questionnaire**

1) This game was:

- Very exciting
- A little bit exciting
- Boring
- Very boring

2) Now you will see some of the cards you chose in the game, mark the answer with an showing how much money you won or lost **altogether** with that card. ONLY ONE OPTION IS CORRECT.

| 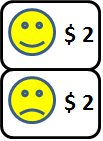 | - I did not win anything - I won $2 - I lost $2 |  | 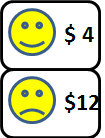 | - I won $4 - I lost $8 - I lost $16 |
| --- | --- | --- | --- | --- |
| 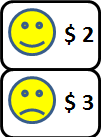 | - I lost $1 - I won $2 - I lost $3 |  | 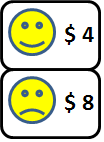 | - I won $4 - I lost $8 - I lost $4 |
| 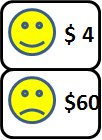 | - I lost $60 - I lost $56 - I won $4 |  | 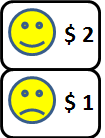 | - I won $3 - I lost $1 - I won $1 |

**YES**

3) Imagine that you will start a new game in which you have to select from two decks, mark in the deck that you would like to choose, and in the deck you wouldn’t like to choose.

**NOO**

- Deck 1: I would gain few money but I would also loses few money too
- - Deck 2: I would gain a lot of money but I would also lose a lot of money too

**Scoring**

1) 4= Very exciting; 3= a little bit exciting; 2= boring; 1= very boring.

2) 1 point for each correct response (range 0-6).

3) 1 point for responding "yes" in the deck 1 and "no" in deck 2.
