# Supplementary material for: Stop Saying That It Is Wrong! Psychophysiological, Cognitive, and Metacognitive Markers of Children’s Sensitivity to Punishment
Source: PLoS One. 2015 Jul 28;10(7):e0133683. doi: 10.1371/journal.pone.0133683 (PMC4517808; doi:10.1371/journal.pone.0133683)
Supplement: S3 Text — (DOCX) [file pone.0133683.s007.docx]

**S3. Cognitive assessment**

**Raven Progressive Matrices Test (RPMT)**

In the RPMT [1] participants completed a series of drawings by considering the spatial organization of an array of objects, identifying relevant features, and choosing one object that matched one or more of the identified features.

**Subtests of working memory from the WISC IV**

We used the three subtests of working memory from the WISC IV [2]: (1) Digit span: [2] consisted of two parts: first, children were required to repeat numbers verbatim as they are stated by the examiner; second, the numbers were repeated in reverse order. The score was calculated from the sum of both parts (maximum 32 points). (2) Arithmetics: included math problems that have to be solved in a time limit (30 sec) (maximum 34 points). (3) Letters and numbers: series of numbers and letters are presented orally and children have to repeat the numbers first (in numerical order), and then the letters (in alphabetical order) (maximum 30 points).

**Hayling test**

In the children’s version of the Hayling test [3, 4] participants are instructed to complete two lists of 15 sentences. In part A, they have to use a word that makes the sentence meaningful, while in part B they have to complete the sentence with a word that does not fit into its context. For example, for the sentence: “*The teacher copied the homework on the ...”* in part A the expected answer is *“blackboard”,* while in part B it is a word that do not make sense with the sentence (e.g. "dog"). In this part, participants have to inhibit the dominant response (the tendency to complete the sentence logically) and use a word unrelated to the syntactic-semantic context of the sentence. We employed the number of errors committed in part B as measure of response inhibition. Thus, zero score was given for responses of unrelated words (correct response), one point for semantically related words, and three points for words that coherently have completed the sentences.

**Trail Making Test (TMT)**

This test [5] consists of two parts in which participants have to connect a set of dots as fast as possible while still maintaining accuracy. The first part (TMT-A) includes numbers and assesses attention and processing speed. The second part (TMT-B) contains numbers and letters and therefore demands cognitive flexibility in order to switch between both categories. The scores are the time (in seconds) in which participants complete the task.

**Battersea Multitask**

The Battersea Multitask Paradigm (BMP) is a children’s test designed to ecologically measure executive functions (EFs) [6]. The BMP includes three interleaved games that participants have to perform within a time limit (three minutes) following four constrained rules. These games include: (1) fruit sorting, participants are instructed to sort small plastic fruits into two small, four medium, and two large boxes; (2) caterpillar coloring, children use crayons to color caterpillars (four short, six medium, and two long) presented on a sheet of paper; (3) counter sorting, children sort counters from a large tub onto grids of varying size: four small, four medium, and two large.

In each game, participants have to sort or color yellow and blue items (fruits, counters, or caterpillar circles) to fill a *cluster* (a box of fruits, a grid of counters, or a caterpillar). Performance is guided by four rules: (1) try the three games before time ends, (2) yellow items get more points than blue ones, (3) full clusters get extra points, and (4) items must be picked up or colored one-by-one. The task goal is to gain points without breaking rules. The optimal strategy is switching between games and fill up small clusters and yellow items first.

Before to start, rules are carefully explained and demonstrated. After that, participants are asked to free recall rules and respond to questions to ensure the understanding of them, e.g., ‘how many games must you try?’. In addition, participants have to generate a plan of how they will perform the task. The planning is self-paced, recorded verbatim, and score according to the following criteria: plan to play the three tasks (0–3 points, 1 point per task), plan to prioritize yellow items (0–3 points, 1 point per task in which prioritizing yellow was planned), and plan to fill up clusters (1 point per task in which filling clusters was planned). The planning score is the sum of these scores (range 0–12 points) and assesses planning skills.

During the performance we computed: (a) the number of tasks attempted (1–3 points, 1 point per task attempted). (b) The strategic performance, reflecting how well the child applied the rules: selecting a yellow item (0–3 points, 1 point for each task), filling smaller clusters (0–6 points: 2 points for a small cluster, 1 point for a medium cluster, 0 points for large cluster), and filling a cluster (0-3 points,1 point for each task in which a cluster was filled before moving on to another cluster; range 1–18 points). (c) Rule breaking, representing the number of errors committed for breaking rules or other mistakes during performance (e.g., yellow item placed into a blue box). The performance composite score includes the number of tasks attempted plus the strategic performance minus the number of errors. This score assesses EFs including cognitive flexibility (task attempted), strategy formation (strategic performance), and inhibitory control (rule breaking).

**References**

1. Raven J, Court J, Raven J, editors. Test de matrices progresivas. Manual. Escalas coloreadas, general y avanzada. Buenos Aires: Paidos; 2008.

2. Wechsler D. Wechsler intelligence scale for children, fourth edition San Antonio: Psychological Corporation; 2003.

3. Burgess PW, Alderman N, Evans J, Emslie H, Wilson BA. The ecological validity of tests of executive function. J Int Neuropsychol Soc. 1998;4(6):547-58. Epub 1999/03/02. PubMed PMID: 10050359.

4. Cartoceti R, Sampedro B, Abusamra V, Ferreres A. Evaluacion de la iniciación y supresión de respuesta verbal en niños . Revista Fonoaudiologica. 2009;52(2):9-24.

5. Spreen O, Gaddes WH. Developmental norms for 15 neuropsychological tests age 6 to 15. Cortex. 1969;5(2):170-91. Epub 1969/06/01. PubMed PMID: 5824433.

6. Mackinlay R, Charman T, Karmiloff-Smith A. High functioning children with autism spectrum disorder: a novel test of multitasking. Brain and cognition. 2006;61:14-24. doi: 10.1016/j.bandc.2005.12.006. PubMed PMID: 16455173.
